# Novel approach to bilateral biliary drainage: EUS-guided hepaticoduodenodenostomy plus hepaticogastrostomy in malignant hilar biliary obstruction

**DOI:** 10.1055/a-2565-8206

**Published:** 2025-04-15

**Authors:** Susumu Hijioka, Yoshikuni Nagashio, Yuta Maruki, Shota Harai, Daiki Agarie, Daiki Yamashige, Kohei Okamoto, Shin Yagi, Soma Fukuda, Yasuhiro Komori, Masaru Kuwada, Yutaka Saito, Takuji Okusaka

**Affiliations:** 1 Department of Hepatobiliary and Pancreatic Oncology, National Cancer Center Hospital, Chuo-ku, Japan; 2 Endoscopy Division, National Cancer Center Hospital, Tokyo, Japan

**Keywords:** Endoscopic ultrasonography, Biliary tract, Intervention EUS, Pancreatobiliary (ERCP/PTCD), Strictures

## Abstract

**Background and study aims:**

Biliary drainage for unresectable malignant hilar biliary obstruction (MHBO) can be technically challenging; therefore, there is a requirement for new treatment methods. This study evaluated the efficacy and safety of a novel bilateral drainage method—endoscopic ultrasound-guided hepaticoduodenostomy plus hepaticogastrostomy (EUS-HDGS)—which combines EUS-guided hepaticoduodenostomy (EUS-HDS) and hepaticogastrostomy (EUS-HGS).

**Patients and methods:**

From 2018 to 2024, we reviewed eight cases of EUS-HDGS from 749 EUS procedures. Both EUS-HDS and EUS-HGS were performed simultaneously. The study outcomes were technical success, clinical success (reduced bilirubin levels or improved cholangitis within 14 days), and adverse events.

**Results:**

Technical success was achieved in 87.5% cases (7/8), whereas clinical success was observed in 75.0% (6/8). Mild peritonitis occurred in 25% of patients (2/8) and 75.0% of patients (6/8) experienced recurrent biliary obstruction, with successful reintervention achieved in all cases. Median stent patency period was 90 days (95% confidence interval: 47.0–133).

**Conclusions:**

EUS-HDGS is a safe and effective drainage method for treating refractory MHBO and has potential as a viable treatment option.

## Graphical Abstract

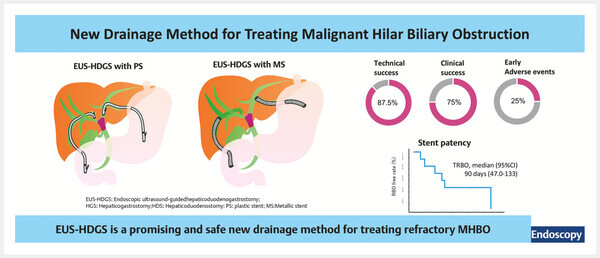

## Introduction

Endoscopic biliary drainage (EBD) via the papilla is recommended for patients with unresectable malignant hilar biliary obstruction (MHBO); however, EBD is technically challenging and often results in failure 1 2 . In particular, in MHBO presenting as Bismuth type II-IV, drainage of an area of 50% or more of the liver volume contributes to overall survival (OS) 3 ; therefore, more effective methods of biliary drainage are required. Previously, percutaneous transhepatic biliary drainage (PTBD) was the standard alternative treatment for cases in which it was difficult to break through the stricture, to do reintervention after metal stent placement, or to approach the papilla.

Endoscopic ultrasound-guided biliary drainage (EUS-BD) has recently attracted attention as a new minimally invasive treatment. Most EUS-BD procedures were initially performed for distal bile duct strictures, and hilar bile duct obstruction was not considered an indication. However, with advances in techniques and devices, there have been an increasing number of reports on drainage of hilar bile duct obstructions using EUS-BD 4 5 6 7 8 9 10 . The main EUS-BD procedures for hilar strictures involve bridging from EUS-guided hepaticogastrostomy (HGS) 11 12 and combining transpapillary drainage with EUS-HGS. However, a new method called EUS-guided hepaticoduodenostomy and hepaticogastrostomy gastrostomy (EUS-HDGS) has become available. This technique combines EUS-guided hepaticoduodenostomy (EUS-HDS) with EUS-HGS into a single procedure. Although there have been several reports of EUS-HDS 9 12 13 , there has been only one case report of EUS-HDGS 14 .

In this study, we aimed to investigate the efficacy and safety of bilateral drainage using EUS-HDGS as a new drainage method for unresectable MHBO.

## Patients and Methods

From 2018 to 2024, our hospital performed a total of 767 cases of interventional EUS. We retrospectively investigated eight cases (0.1%) that underwent EUS-HDGS. This study was conducted in accordance with the principles of the Declaration of Helsinki and was approved by the Ethics Committee of the National Cancer Center, Japan (2018–149).

**Fig. 1:**
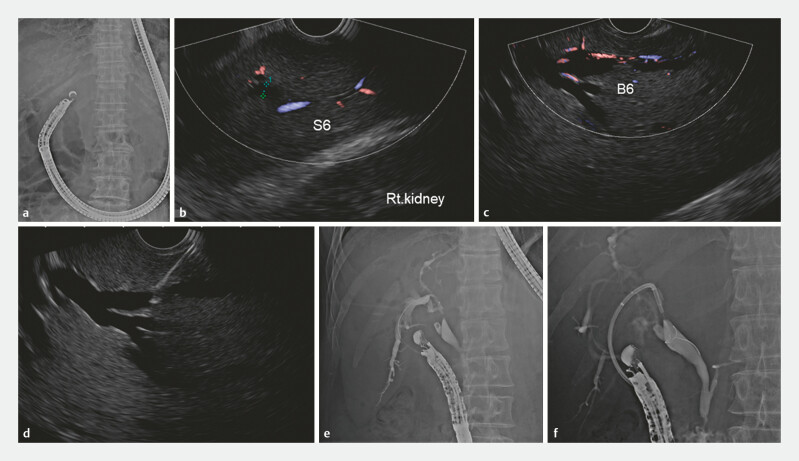
Method of endoscopic ultrasound-guided hepaticoduodenostomy (EUS-HDS). **a** The EUS scope is positioned longitudinally, forming a U-shape. **b** The right kidney was confirmed in the EUS image as a B6 anatomical landmark. **c** The B6 puncture bile duct is drawn so that the puncture angle is obtuse. **d** EUS image punctured by 19G needle. e The guidewire and contrast were inserted up to the hepatic hilar. f A plastic stent is placed to complete the hepaticogastrostomy.

The EUS-HDS procedure involved inserting the echoendoscope (UCT-260; Olympus, Tokyo, Japan; EG-740UT; FUJIFILM Medical, Tokyo, Japan) into the duodenal bulb. In the duodenal bulb, the EUS scope was positioned in a U shape to visualize the portal vein and bile duct of the hilar lesion ( Fig. 1 ). Then, the scope was rotated clockwise, following the right portal vein and the right intrahepatic bile duct. Anatomical landmarks for the posterior segment include the right hepatic vein and the right kidney. In addition, the fluoroscopic position of the scope was referenced. The dilated bile duct was differentiated from the portal vein using color Doppler.

The B6 bile duct was selected as the primary puncture target because it allows for an obtuse puncture angle and facilitates guidewire advancement toward the hepatic hilum. However, if the gallbladder or the portal vein obstructs access to B6, the anterior segment bile duct can be punctured instead. Because risk of bile leakage increases when the distance to the target bile duct is <2.5 cm 15 , we carefully considered liver parenchymal thickness between the puncture site and the bile duct, and the puncture point was selected to maximize this distance whenever possible. The appropriate needle was selected after the target bile duct for puncture was determined. If the bile duct diameter is ≥2.5 mm, a 19G fine-needle aspiration (FNA) needle should be used; however, if it is <2.5 mm, a 22G FNA needle should be chosen. After puncturing the bile duct, cholangiography was performed, and the guidewire was inserted: a 0.025-inch guidewire for 19G punctures and a 0.018-inch guidewire for 22G punctures.

**Fig. 2:**
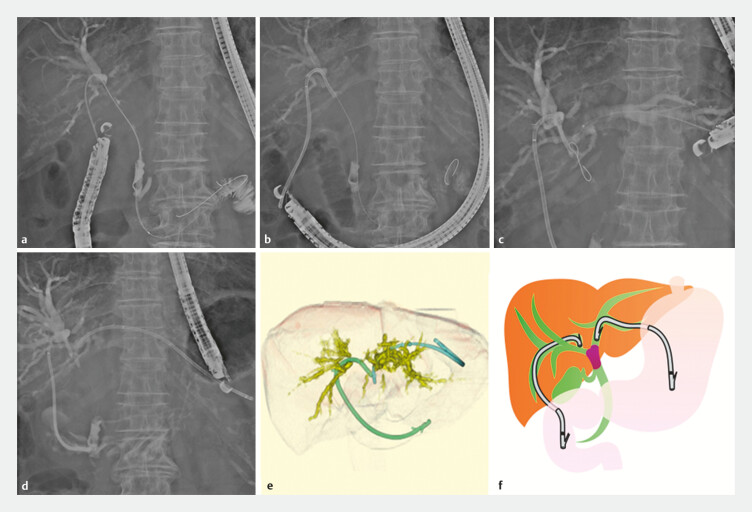
Case of endoscopic ultrasound-guided hepaticoduodenostomy and hepaticogastrostomy (EUS-HDGS) with a plastic stent (PS). **a** Puncture with a 19G needle and contrast from the duodenal bulb to the posterior bile duct. **b** A PS is placed to complete the hepaticoduodenostomy. **c** A guidewire is inserted from the stomach to the left lobe bile duct (B3). **d** A PS is placed to complete the hepaticogastrostomy. **e** Three-dimensional image at the time of completion. **f** EUS-HDGS schematic.

**Fig. 3:**
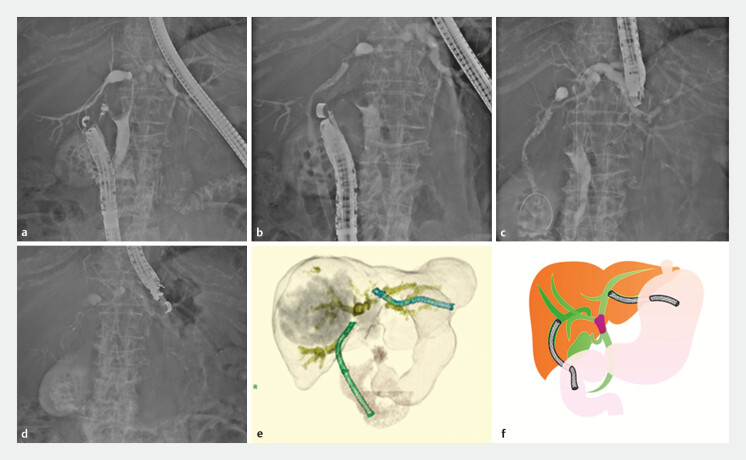
A case of endoscopic ultrasound-guided hepaticoduodenostomy and hepaticogastrostomy (EUS-HDGS) with a metallic stent (MS). **a** The guidewire is inserted from the duodenal bulb to the posterior bile duct. **b** A MS is inserted to complete the hepaticoduodenostomy. **c** The guidewire is inserted from the B3 to the duodenum. **d** A MS is placed to complete the hepaticogastrostomy. **e** Three-dimensional image at the time of completion. **f** EUS-HDGS schematic.

Mechanical dilators or drill dilators were primarily used for fistula dilation to minimize bile leakage. If stent placement remained difficult, balloon dilation up to 4 mm was performed before placing the stent. A cystotome was not used. During the same session, a stent was placed in the left intrahepatic bile duct (HGS) by puncturing through the stomach. Regarding use of metal stents (MSs) or plastic stents (PSs) in the earlier period, a MS was used in all patients. Later, MSs were selected when there was tumor thrombosis, bleeding due to tumor invasion of the bile duct, and ascites, whereas a PS was selected in other cases ( Fig. 2 and Fig. 3 ).

All cases in this study underwent EUS-HDS and EUS-HGS in a single session, without an interval between the two procedures. Procedure duration was defined as time from puncture of the right intrahepatic bile duct to completion of stent placement for both drainage routes ( **Supplementary video** ).

### Definitions of outcomes

Main outcomes included technical success rate, clinical success rate, and incidence of adverse events (AEs). Technical success was defined as successful stent placement in the intended location before the procedure for both HDS and HGS. Clinical success was defined as a decrease in total bilirubin level to <50% of the previous value, normalization within 14 days, or improvement in cholangitis. Procedure time was defined as time from HDS puncture to HGS stent placement. AEs were graded according to the severity grading system of the American Society for Gastrointestinal Endoscopy lexicon 16 . Recurrent biliary obstruction (RBO) was defined as obstruction of either the HDS or HGS stent, as determined by clinical symptoms (jaundice, cholangitis) and imaging findings. Time to RBO (TRBO) was determined according to the Tokyo Criteria 2024 17 . Follow-up evaluations were performed regularly using blood tests and imaging studies.

### Statistical analysis

Continuous variables are expressed as medians (ranges) and categorical variables are expressed as numbers (percentages). Qualitative differences between groups were evaluated using Fisher's exact test for categorical parameters. TRBO with 95% confidence intervals (CIs) were calculated using the Kaplan-Meier method, and patients were censored when on the last day of follow-up or death before RBO. All *P* values were two-sided, and *P* < 0.05 was considered statistically significant. All statistical analyses were performed using SPSS version 29.0 (IBM Corp., Armonk, New York, United States).

## Results

### Patient baseline characteristics

Median age of the eight patients was 69 years (range 52–83), and five patients (62.5%) were male ( Table 1 ). Primary diseases were bile duct cancer in six patients (75.0%), hepatocellular carcinoma (HCC) in one patient (12.5%), and colorectal cancer in one patient (12.5%). Bismuth classification was type II/III/IV in two (25.0%), five (62.5%), and one (12.5%) patients, respectively. Timing of drainage was rescue in all cases. In the last procedure before EUS-HDGS, the PS and MS were used in six (75.0%) and two (25.0%) cases, respectively.

**Table:** **Table 1** Patient characteristics.

Analysis cohort	Patients N = 8
Age, years (y, range)	69 (52–83)
Sex, male	5 (62.5%)
Primary disease	
Bile duct cancer	6 (75.0%)
Hepatocellular carcinoma	1 (12.5%)
Colon cancer	1 (12.5%)
Bismuth classification	
Bismuth type 2	2 (25.0%)
Bismuth type 3	5 (62.5%)
Bismuth type 4	1 (12.5%)
Timing of drainage	
Rescue drainage	8 (100%)
Type of stent used in the last procedure	
2 Plastic stent	4 (50.0%)
3 Plastic stent	2 (25.0%)
3 Metallic stent	2 (25.0%)

### Clinical outcomes

Results and details of the procedure are shown in Table 2 .

**Table:** **Table 2** Procedure detail.

Analysis cohort	Patients N = 8
Technical success	7 (87.5%)
Clinical success	6 (75.0%)
Median procedure time (m, range)	57 (41–117)
HDS procedure	
Puncture site (n, %)	
Posterior	6 (75.0%)
Anterior	1 (12.5%)
B4	1 (12.5%)
Distance from the puncture site to bile duct	2.8 cm (range: 1.6–3.5 cm)
Type of stent (n, %)	
PS (7F)	2 (25.0%)
PS (6F)	1 (12.5%)
MS (6 mm)	1 (12.5%)
MS (8 mm)	4 (50.0%)
Method of fistula dilatation (n, %)	
Mechanical dilator only	2 (25.0%)
Drill dilator only	2 (25.0%)
Balloon catheter (additional)	4 (50.0%)
HGS procedure	
Puncture site (n, %)	
B2	2 (25.0%)
B3	6 (75.0%)
Distance from the puncture site to bile duct	3.0 cm (range: 2.0–4.1 cm)
Type of stent (n, %)	
PS (7F)	3 (37.5%)
MS (6 mm)	1 (12.5%)
MS (8 mm)	4 (50.0%)
Method of fistula dilatation (n, %)	
Mechanical dilator only	2 (25.0%)
Drill dilator only	3 (37.5%)
Balloon catheter (additional)	3 (37.5%)
Recurrent biliary obstruction	
HDS side	2 (22.2%)
HGS side	3 (37.5%)
Adverse events	
Peritonitis(mild)	2 (25.0%)
Reintervention (n=5)	
Technical success	5 (100%), n = 5
HDS, hepaticoduodenostomy; HGS, hepaticogastrostomy; MS, metal stent;PS, plastic stent.	

Technical and clinical success rates were 87.5% (7/8) and 75.0% (6/8), respectively. Puncture sites for HDS were the posterior region at 75.0% (6/8) and the anterior region at 12.5% (1/8). One patient experienced technical failure due to a bile duct puncture during HDS, and a stent was placed in B4 using the Couinaud classification.

Stents placed during the HDS and HGS procedures were PS and MS in three (37.5%) and five (62.5%) cases, respectively. Median total procedure time was 57 minutes (range 41–117).

Two (25.0%) cases had early AEs, both of which were mild peritonitis. No stent migration or displacement occurred within 14 days of surgery.

**Fig. 4:**
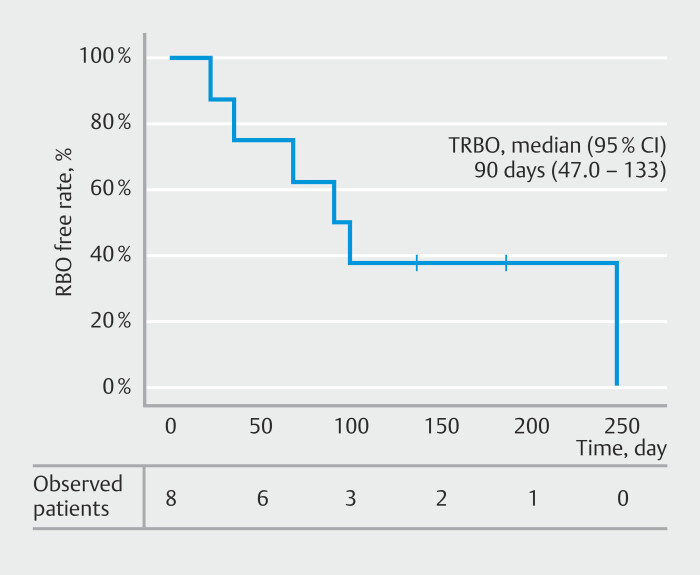
Time to recurrent biliary obstruction. Median stent patency period was 90 days (95%CI 47.0–133).

RBO was observed in six patients (75%), reintervention was performed in five cases, and stent replacement was possible in all cases. Median stent patency period was 90 days (95% CI 47.0–133) ( Fig. 4 ).

## Discussion

A case of endoscopic ultrasound-guided hepaticoduodenostomy and hepaticogastrostomy (EUS-HDGS) for hepatocellular carcinoma. A metallic stent (MS) was selected because of tumor invasion of the bile duct. A MS with a diameter of 6 mm and a length of 12 cm was used.Video 1

Bilateral drainage with EUS-BD for hilar obstruction is a new method that has attracted attention in recent years. The main technique comprises EUS-HGS with the bridging method and EUS-BD with transpapillary drainage (combination method). We previously reported on EUS-HDGS, which involves drainage of both the left and right lobes in the same session. This is the first case report of eight consecutive cases in which EUS-HDGS was attempted. The technical success rate was 87.5%. The procedure was unsuccessful due to failure on the HDS side and a bile duct puncture error in B4. The cause was thought to be insufficient understanding of the anatomy of the right lobe of the liver from the duodenal bulb. As a solution, we consistently obtained a right anterior oblique view after bile duct puncture to confirm that it was the target bile duct branch before stent placement.

HDS puncture is mainly performed in the posterior duct but can also be performed in the anterior branch. In cases such as Bismuth type II or IIIb, where drainage to either the anterior or posterior region is acceptable, it is best to choose the side that is easier to puncture, avoiding the gallbladder and blood vessels aiming for placement where the guidewire is more likely to face the hepatic hilum.

The main advantage of HDGS is that it can divide the bile duct into two drainage routes (three if the transpapillary route is included). It is considered a good option for refractory cases that repeatedly develop cholangitis after insertion of multiple PSs or MSs via the transpapillary route. In particular, cases of bile duct invasion by HCC ( Fig. 3 ) were very effective because it was possible to perform drainage without passing through the tumor.

Regarding selection of PS or MS, the PS generally has a higher risk of bile leakage than the MS; however, we have taken several measures to minimize bile leakage, such as using minimal dilation and ensuring adequate liver parenchyma retention at the puncture site. Furthermore, based on our previous retrospective study on EUS-HGS 18 , we found that PSs were associated with fewer complications and were safer overall compared with MSs. Consequently, we primarily used PSs in the latter half of this study. In fact, the two cases of peritonitis in our study occurred in MS cases, whereas no peritonitis was observed in PS cases.

However, for cases with ascites or bile duct invasion, in which it is best not to touch the tumor with the stent, we prioritized MSs ( Video 1 ).

Another advantage of stenting by EUS-HDGS is that it does not cause cholecystitis or pancreatitis, which sometimes occurs with hilar drainage.

Regarding AEs, we encountered two cases of peritonitis. In both cases, time was required to dilate the bile duct of the posterior branch by balloon. Because the long position makes applying force when inserting the device more difficult, the cause is bile leakage after dilation. Countermeasures include bile aspiration without dilation 19 20 and bile aspiration using a catheter 21 . TRBO tends to have a shorter patency period compared with that in normal HGS. This is because two stents are present, and we consider this unavoidable. However, reinterventions were successful in all cases (5/5 cases, 100%), and ease of replacement is also one of the features of HDGS.

This study is limited by its retrospective design and the small sample size from a single institution, which may impact generalizability of our findings. In addition, median stent patency period (90 days) provides only a preliminary estimate of long-term durability, and larger-scale studies with extended follow-up are needed to validate our findings. However, MHBO is a condition encountered relatively frequently in clinical practice, and effective biliary drainage remains a critical challenge. EUS-HDGS has the potential to provide an alternative treatment strategy in complex cases. Despite the small sample size, this study provides valuable preliminary data demonstrating its feasibility, safety, and efficacy.

In addition, the bilateral drainage technique is technically demanding and may not be easily reproducible in centers with a low volume of EUS-guided biliary drainage cases. Although this remains a challenge, it is important to note that the fundamental procedure steps, including EUS-HDS and HGS, have been well-established in expert centers. As experience with these techniques grows, standardization of procedure strategies and training programs will likely improve reproducibility across different institutions.

## Conclusions

We are currently accumulating more cases and planning prospective studies to further validate our findings and facilitate the broader adoption of EUS-HDGS. These efforts will be essential in determining the long-term outcomes and the learning curve associated with this approach.
